# *Pseudomonas aeruginosa* Scleritis following Pterygium Surgery with Mitomycin C or Beta Irradiation: Three-Case Report

**DOI:** 10.1155/2022/8000944

**Published:** 2022-05-05

**Authors:** Winai Chaidaroon, Somsanguan Ausayakhun, Napaporn Tananuvat, Phit Upaphong, Kittipong Thabsuwan

**Affiliations:** Department of Ophthalmology, Faculty of Medicine, Chiang Mai University, Chiang Mai 50200, Thailand

## Abstract

**Purpose:**

To report three cases of culture-positive *Pseudomonas aeruginosa* scleritis following pterygium surgery. *Patients and Methods*. A retrospective study of all patients of *Pseudomonas aeruginosa* scleritis after pterygium surgery presented from May 2017 to May 2020 was performed. Patient demographics and clinical characteristics included age, gender, time between prior surgery and onset, adjunctive therapy, risk factors, initial visual acuity, final visual acuity, clinical features, medical treatment, and surgical intervention were analyzed.

**Results:**

Three eyes of three patients with clinical characteristics and laboratory-confirmed *Pseudomonas aeruginosa* scleritis were identified. Two patients were related with mitomycin C application after pterygium surgery, and only one had beta irradiation. Antibiotic administration and scleral debridement were required in 3 patients. One eye was enucleated. Final visual outcomes of two patients were improved.

**Conclusions:**

*Pseudomonas aeruginosa* scleritis after pterygium surgery is a crucial ophthalmic disease. An early diagnosis with a prompt intensive antibiotic treatment in combination with surgical interventions may improve visual outcome.

## 1. Introduction

Scleritis, an inflammatory ocular disease of the sclera, is a devastating disorder that can cause permanent visual loss [1]. It has a diversity of clinical manifestations and causative factors. The major etiology is the immunological origin. However, scleritis caused by infections accounts for 5-10%. Infectious scleritis may occur after accidental or surgical injuries, severe endophthalmitis, or primary corneal infection, which can cause several vision-threatening complications such as cataract, glaucoma, and loss of the eye [2, 3]. Among a variety of organisms that have been isolated from infectious scleritis, *Pseudomonas aeruginosa* remains the most common infectious pathogen [4].

Pterygium is an ocular surface condition manifested by a triangular-shaped fibrovascular formation arising from the conjunctiva onto the cornea [5, 6]. A widely accepted risk factor for this disease is prolonged exposure to ultraviolet or sunlight, which damages the limbal stem cell barrier [7]. Thus, pterygium excision is one of the most common ophthalmic operations in patients who live in tropics and subtropics [2]. The bare sclera technique is the first surgical intervention associated with high rates of pterygium recurrence and postoperative infection [8]. *Pseudomonas aeruginosa* scleritis is commonly associated with the excision of pterygium and may occur years after surgery.

The main objective of this study is to report three cases of *Pseudomonas aeruginosa* scleritis following pterygium excision. The clinical characteristics, previous surgery with adjunctive treatments, treatments (medical and surgical therapy), and visual outcomes are described.

## 2. Patients and Methods

A retrospective descriptive study was conducted on the computer-encoded medical records of patients who had culture-proven *Pseudomonas aeruginosa* scleritis at the Ophthalmology Clinic, Chiang Mai University Hospital. The study protocol was conducted in accordance with the tenets of the Declaration of Helsinki, and the protocol was approved by the Ethics Committee of the Faculty of Medicine, Chiang Mai University (Study code: OPT-2564-08608). Written informed consents were obtained from all patients before the publication. In order to find recent cases of this rare disease, the database of medical records of clinical case summaries between May 2017 and May 2020 was cross-referenced against computer-coded cases with a diagnosis of *Pseudomonas* scleritis. Three patients (3 infected eyes) were identified. The computer records containing patient demographics and clinical characteristics were downloaded and analyzed. These data included age, gender, the time between prior surgery and onset, adjunctive therapy, initial visual acuity, final visual acuity, clinical features, medical treatment, and surgical intervention. Clinical notes for cases 1–3 were demonstrated. The summaries of all cases have been included in this report.

## 3. Case Presentation

### 3.1. Case 1

A 67-year-old male was referred to the tertiary hospital with severe pain, redness, and blurred vision in his right eye. He had a 12-year history of pterygium excision with the mitomycin C eye drops. At a secondary hospital, the doctor treated him as necrotizing scleritis with 0.5% moxifloxacin eye drops hourly in the right eye and ciprofloxacin (500 mg) one tablet twice daily for seven days without improvement before referral.

**Figure 1:**
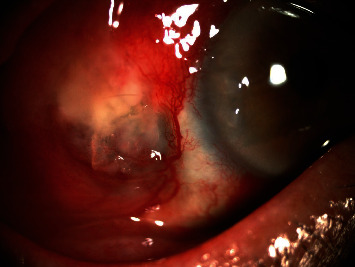
Slit-lamp photograph of the right eye of case 1, necrotic scleral thinning surrounded by edematous necrotic tissue was found at the temporal sclera near the limbus.

On his initial examination, the best-corrected visual acuity (BCVA) was 6/36, OD and 6/12, OS and marked injection of the conjunctiva with severe mucopurulent discharge. Slit-lamp biomicroscopy revealed necrotic scleral thinning surrounded by edematous necrotic tissue at the temporal sclera near the limbus in the right eye without anterior chamber reaction (Figure 1). The cornea was normal. Fundoscopic examination showed normal fundus. The left eye had a mild cataract, and the fundoscopy was also normal. The intraocular pressures were normal in both eyes. Scraping was obtained from the necrotic sclera and the base of the active lesion for microscopic examination and culture. The result of bacterial culture revealed *Pseudomonas aeruginosa* which was sensitive to amikacin, colistin, ceftazidime, imipenem, meropenem, ciprofloxacin, and levofloxacin. The patient was admitted to the hospital, and initial treatment was initiated with fortified amikacin (20 mg/mL) eye drops hourly and intravenous ceftazidime 1 g every eight hours. After four days of unresponsive medical treatment, superficial necrotic scleral tissue debridement was performed. The BCVA improved from 6/36 to 6/12 in the right eye, with an improvement in scleral thinning three weeks after admission. Fortified amikacin (20 mg/mL) eye drops were tapered within one month and replaced with levofloxacin eye drops four times daily. The BCVA of the right eye was 6/12, and the scleral lesion was healed completely without further thinning two months after treatment.

### 3.2. Case 2

A 68-year-old female, with a history of previous pterygium excision with mitomycin C application in the right eye five years ago, was referred to the ophthalmology clinic with right eye pain, redness, and decreased vision for three days. She was treated by a general ophthalmologist with 0.3% gentamicin eye drops hourly for a few days, but increased pain and redness and decreased vision occurred. She denied a history of systemic diseases.

**Figure 2:**
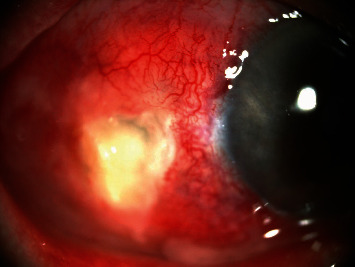
Slit-lamp photograph of the right eye of case 2 showed severe congestion of temporal conjunctival vessels, mucopurulent discharge, and necrotic scleral ulcer in the area of previous pterygium excision.

On eye examination, the BCVA was 6/18, OD and 6/9, OS. Slit-lamp biomicroscopy of the right eye showed a necrotic scleral ulcer in the area of previous pterygium excision and marked congestion of temporal conjunctival vessels with severe mucopurulent discharge. No hypopyon was found (Figure 2). The intraocular pressure and fundoscopy were normal. The fellow eye was normal.

Scleral specimens collected from the scleral ulcer disclosed many Gram negative bacilli and culture yielded *Pseudomonas aeruginosa*, which was sensitive to amikacin, gentamicin, ceftazidime, imipenem, meropenem, ciprofloxacin, and levofloxacin. As *Pseudomonas aeruginosa* scleritis was a definite diagnosis, the empirical treatment was initiated with topical fortified gentamicin (14 mg/mL) and cefazolin (33 mg/mL) eye drops hourly. Superficial necrotic scleral tissue debridement was performed. After four days, the frequency of eye drops was gradually decreased to every two hours with clinical improvement. Intravenous amikacin and ceftazidime were administered for three days. After four weeks of admission, the scleral ulcer turned to dark discoloration with less inflammation. The patient was discharged with BCVA of 6/6 in the right eye.

### 3.3. Case 3

**Figure 3:**
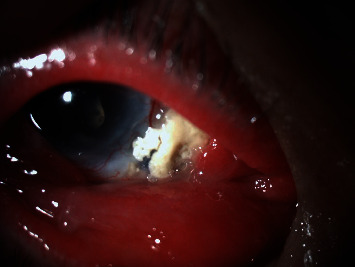
Slit-lamp photograph of the right eye of case 3 demonstrated severe eyelid swelling, marked chemosis, and scleral necrotic scleral ulcer size of 3 × 4 mm with calcified plaques at the nasal side of the previous pterygium excision.

A 70-year-old male had a history of right nasal pterygium excision with bare sclera technique and beta irradiation application 25 years ago. He also had a history of complicated cataract surgery in his right eye for five years prior to this admission. He presented at our hospital with severe right eye pain, blurred vision, redness, and swelling of the eyelids for seven days. With uncontrolled diabetes mellitus, the fasting blood sugar was 200 mg/dL and hemoglobin A1c was 7.1%. At presentation, the BCVA was hand movement, OD and 6/12, OS. Slit-lamp biomicroscopy of the right eye revealed conjunctival hyperemia, severe eyelid swelling, marked chemosis, and necrotic scleral ulcer size of 3 × 4 mm with calcified plaques at the nasal side of the previous pterygium excision (Figure 3). Mild corneal opacity was found without hypopyon. The fundoscopy showed severe vitritis that obscured fundus findings. Examination of the left eye was normal. He was admitted for diabetic monitoring and proper eye treatment. He was brought to the operating room for microbial work up on the scleral site and the vitreous after a well glycemic control. The necrotic scleral tissues were debrided with ceftazidime irrigation. Vitreous tapping and a combination of intravitreal ceftazidime and vancomycin injection were performed. The specimens from vitreous, and sclera exhibited heavy Gram negative rod organisms. The culture findings illustrated *Pseudomonas aeruginosa*. The initial topical therapy was started with fortified amikacin (20 mg/mL) eye drops hourly and intravenous ceftazidime 1 g every eight hours. Four days later, the patient suffered severe eye pain with no light perception. He preferred to have his right eye removed by an enucleation procedure after a lengthy discussion.

Patient demographics and clinical characteristics are summarized in Table 1.  Table 2 illustrates the antimicrobial susceptibility of *Pseudomonas aeruginosa* obtained from scleral tissues determined by using microdilution with microblot assay.

## 4. Discussion

*Pseudomonas aeruginosa* scleritis is a rare disorder that may cause marked thinning and melting of the sclera, leading to endophthalmitis, and the resulting loss of vision. Many studies revealed that advanced age may be a contributing factor to *Pseudomonas* scleritis [9–11]. Additionally, in a large retrospective study of infectious scleritis, Hodson et al. found that the median age of the patients who suffered from infectious scleritis was 70 years [12]. All cases in our study were over 60 years old. These findings may reflect that age could be one of the risk factors for *Pseudomonas aeruginosa* scleritis or the nature of a certain latency period after pterygium excision. *Pseudomonas aeruginosa* was detected in diabetic subjects more predominantly than nondiabetic subjects, demonstrated by the study of Noche et al. [13]. One patient in our study also had diabetes that may be a risk factor for *Pseudomonas aeruginosa* scleritis. Nuzzi and Tridico showed that there was a high recurrent rate of pterygium after simple pterygium excision [14]. Consequently, adjunctive therapy such as mitomycin C and beta irradiation had been used to prevent the recurrence. In this study, two patients had clear histories of mitomycin C and only one had beta irradiation application as the adjuvant therapy for at least 20 years. *Pseudomonas aeruginosa* scleritis following pterygium surgery with mitomycin C and beta irradiation had been proposed [15–17]. Several reasons suggested by Meallet indicated that the application of either mitomycin C or beta irradiation was inclined to compromise conjunctival blood vessels and subconjunctival connective tissues, thus restraining normal conjunctival wounding healing and leaving bare sclera for facile infection [18]. Bare sclera without intact conjunctival epithelium may be susceptible to infection. *Pseudomonas aeruginosa* employs neutrophil-activated collagenases to damage ocular tissues, in particular, compromised sclera [19]. Moreover, *Pseudomonas* bacteria could produce extracellular proteases. These destructive enzymes may contribute to the extension of subconjunctival *Pseudomonas* scleritis [2]. An animal experiment study by Kessler et al. indicated that the destruction of ocular tissue by *Pseudomonas* involved the protease produced by *Pseudomonas* and host response of both collagenolytic and proteoglycanolytic enzymes [20]. The duration between the previous surgical procedure and the onset in our study varied from 5 to 25 years. Alsagoff et al. and O'Donoghue et al. reported that the longest latent period was 40 years [21, 22]. The explanation for such a long latent period was undetermined; however, systemic autoimmune diseases or the surgery itself could stimulate or modify the hypersensitivity reaction associated with pathogens [23].

The clinical characteristics of *Pseudomonas aeruginosa* scleritis usually exhibit localized scleral inflammation and necrosis. In this study, two patients had severe pain and necrotic scleral melting consistent with typical *Pseudomonas* scleritis. Another was a diabetic patient with a severe scleral infection and endophthalmitis at presentation.

Initial management of *Pseudomonas aeruginosa* scleritis in our study included topical fortified antibiotics in all cases. Huang et al. reported that early surgical debridement and antibiotic irrigation should be highly encouraged in patients with severe scleritis which was poorly responded to aggressive medical treatment [2]. Regarding surgical intervention, three eyes in our study had undergone debridement of necrotic scleral tissue. Surgical debridement not only had a good expedition of antibiotic penetration but also removed the volume of infected necrotic tissues. A unique collagen fiber arrangement of the sclera and a less vascular layer of an outer coat of the eye causes poor penetration of antibiotics for *Pseudomonas* scleritis treatment. A previous study confirmed the persistence of *Pseudomonas* pathogen despite laborious antibiotic treatment [17]. However, one eye in our study did not respond to both intensive antibiotic therapy and surgical interventions, which may be related to a strong virulence of *Pseudomonas aeruginosa* and/or host immune response to the microorganisms. The literature reviewed by Reynolds and Alfonso showed that nearly 60% of intensively treated eyes had undergone evisceration or enucleation [24]. Interestingly, all cases in this study had clinical improvement without corticosteroid treatment, except one case that had diabetes with severe endophthalmitis. The role of treatment with antibiotics and corticosteroids in ocular *Pseudomonas aeruginosa* infection remains uncertain [25, 26]; thus, we agreed not to use corticosteroids in three cases, especially in case 3 who had diabetes.

## 5. Conclusion

We highlighted three cases of *Pseudomonas aeruginosa* scleritis following pterygium surgery with the history of the application of either mitomycin C or beta irradiation. Early diagnosis with rapid intensive antibiotic treatment in conjunction with surgical interventions may improve visual outcomes.

## Figures and Tables

**Table 1:** Demographics, clinical characteristics, treatments, and outcomes of three cases with *Pseudomonas aeruginosa* scleritis.

Case	Sex/age (yr)/eye	Adjunctive therapy	Latent period (yr)	Associated risk factors	BCVA initial-final	Clinical features	Treatments	Additional procedures
1	M/67/OD	Mitomycin C	12	None	6/36-6/12	Scleral thinning surrounded by edematous necrotic tissue	Fortified amikacin eye drops and intravenous ceftazidime	Superficial necrotic scleral debridement
2	F/68/OD	Mitomycin C	5	None	6/18-6/6	Necrotic scleral ulcer with severe mucopurulent discharge	Fortified gentamicin and cefazolin and intravenous amikacin and ceftazidime	Superficial necrotic scleral debridement
3	M/70/OD	Beta irradiation	25	Diabetes mellitus	Hm	Severe lids swelling, marked chemosis, scleral ulcer with calcium plaques	Fortified gentamicin and cefazolin and intravenous amikacin and ceftazidime	Vitreous tapping with intravitreal antibiotics injection and scleral debridement with ceftazidime irrigation Enucleation

BCVA: best-corrected visual acuity; F: female; Hm: hand movement; M: male; OD: right eye; yr: year.

**Table 2:** Drug sensitivity patterns of *Pseudomonas* isolates of three cases by microdilution with microblot assay.

Drug	Case 1	Case 2	Case 3
Gentamicin Cefepime Amikacin Ciprofloxacin Colistin Doripenem Ceftazidime Imipenem Levofloxacin Piperacillin-tazobactam Meropenem	S S S S S S S S S S S	S S S S R S R S S R S	R R S R R R R I R I R

I: intermediate; R: resistant; S: sensitive.

## Data Availability

All data are available upon request.
